# The Duties of State Assayers in Relation to Quack Medicines

**Published:** 1857-12

**Authors:** 


*The Duties of State Assayers in Relation to Quack Medicines.*—The newspapers are constantlypuffing quack medicines, whose innocuous or beneficial effects are certified to by State Assayers. One of the most unprofessional of these certificates accompanies the Peruvian Syrup. Whatever the wording may be, the readers of quack advertisements infer, from its tone, that the Peruvian Syrup has virtues which are not possessed by the citrate, tartrate, lactate and other preparations well known to the profession. There is a common idea in the public mind, that the tincture of the chloride, and iron rust, are the forms in which Iron is given, and that this certificate is in favor of a substitute for those exceedingly disagreeable preparations. To the profession at large, the Peruvian Syrup is a secret remedy, and as a secret remedy it does harm. Is not the certificate in favor of such irregular practice?

Every paper that comes to your door contains a State Assayer's puff for somebody's Bourbon whiskey. Can a State Assayer's analysis show the difference between Columbia and Bourbon?

Green's mixture of cinchona and sulphuric acid used to bear an assayer's certificate, if it does not now; an irregularity which, in any other State, would subject him to trial before the medical association.

It has been reported quite extensively, that a State Assayer's duties under the law require that he should give a certificate of the composition of any article brought to him for examination. The only statute which I can find (there may be others) in the Massachusetts laws, alluding to the duties of that office, is as follows:

"§ 1. *Be it enacted, &c.* The Governor, with the advice and consent of the Council, may appoint one or more suitable persons to be assayers of ores and metals, who shall be sworn to the faithful discharge of their duties.

"§ 2. It shall be the duty of each assayer to assay such ores and metals as may be offered him for assay, and to give a certificate thereof, for which service he shall be paid a reasonable compensation by the person procuring such assay to be made.

"Approved March 18th, 1846."

It must be under the provisions of some other act that the assay of Bourbon Whiskey and Peruvian Syrup come, but I cannot find it.

The object of making the office doubtless was, to encourage the working of valuable mineral deposits, which were known or supposed to exist in various parts of the State. The manufacture of quack medicines is a branch of industry, probably, not in the view of the Legislature of 1846. —*Boston Medical and Surgical Journal.* C. E. B.

---

*Simple Method of Preventing Accidents from Chloroform.*—The following plan is recommended by a correspondent of the Medical Times and Gazette, as one which the author has found uniformly successful both in midwifery and surgical cases. He says:

Although I have used it at least one thousand times, I have never seen the least bad consequences follow from it, and I consider that this success depends greatly on the precaution I take before administering the chloroform; this simply consists in administering a glass of spirits or wine—I prefer the former, even to ladies. The wine, or spirits, seem to exercise no effect on the chloroform, while their stimulating quality keeps up the action of the heart during the time the patient is under chloroform, and prevents sinking. I had occasion some years ago, to perform a slight surgical operation on a lady who was fearfully afflicted with asthma and excessively nervous. Her husband being a medical man, now in the west-end of London, objected to the use of chloroform in such a case, but I assured him that the wine would prevent any evil happening. The operation was performed, the patient saved from the pain of it, and to her great relief she had no return of asthma for a long time, and when it did return, she had recourse to the chloroform, which, for a time, gave her great relief. On one occasion, while I was removing a scirrhous tumor, the patient, who was rather advanced in life, got an overdose of chloroform, and we had great fears of her being permanently roused, and I do believe her recovery was owing entirely to injecting a glass of brandy and water into the rectum. The accident happened owing to the gentleman who had charge of the chloroform getting so interested in the dissection, that he forgot to raise the towel off her face till respiration had become imperceptible. However, she soon rallied.—*Medical Times & Gaz.; Braithwaite's Retrospect*, 1857, from *N. O. Med. & Surg. Journal.*

